# Moral self-concept and moral sensitivity in Iranian nurses

**Published:** 2015-04-04

**Authors:** Fariba Borhani, Mohammad Keshtgar, Abbas Abbaszadeh

**Affiliations:** 1 Associate Professor, Medical Ethics and Law Research Center, Shahid Beheshti University of Medical Sciences, Tehran, Iran;; 2 MSc in Nursing, Nursing Department, Medical Ethics and Law Research Center, Shahid Beheshti University of Medical Sciences, Tehran, Iran;; 3 Professor, Department of Medical-Surgical Nursing, School of Nursing and Midwifery, Shahid Beheshti University of Medical Sciences; and Academy of Medical Sciences, Tehran, Iran.

**Keywords:** moral self-concept, moral sensitivity, moral decision making, nurse, Iran

## Abstract

Nurses are often faced with serious situations that require high levels of legal and ethical knowledge, and should therefore be sensitive to the moral issues in their profession in the decision making process. Some studies have investigated nurses’ moral self-concept as an effective factor in moral sensitivity, but there is not sufficient evidence to support this. The purpose of this study was to determine the correlation between moral sensitivity and moral self-concept in nurses employed in the teaching hospitals in Zahedan, Iran.

This cross-sectional descriptive study aimed to study the relationship between moral self-concept and moral sensitivity in nurses employed in the teaching hospitals affiliated with Zahedan University of Medical Sciences. Chang’s Moral Self-Concept Questionnaire and Lutzen’s Moral Sensitivity Questionnaire were used for data collection. Data analysis was performed using SPSS software version 17.

A total of 188 nurses participated in this study. The results showed that there was a positive and significant relationship between moral self-concept and moral sensitivity (*P* < 0.05).

Based on our findings, an individual's attention to moral issues can lead to greater sensitivity and result in morally responsible behavior at the time of decision making. Consequently, promotion of moral self-concept through personal effort or education can increase moral sensitivity, which in turn leads to behavioral manifestations of ethical knowledge.

## Introduction

In the past two decades, opportunities resulting from developments in bioethics have been provided all around the world (1). The ever-increasing scientific progress has posed important moral questions to the human society that need to be answered. In medicine, there have been major developments in technology and treatment methods, creating progressively more complicated moral issues in patient care. Nurses, who comprise a substantial group of health service providers, are naturally not excluded from this phenomenon (2).

Changes in nursing concepts and health needs, end of life care, increasing numbers of patients, changes in nursing values, improvements in medical technology, resource allocation, rising costs, population ageing, and greater attention to individual rights have caused new moral conflicts that nurses must face on a daily basis (3, 4). In order to solve these problems, nurses must gain skills and abilities in moral decision making (5, 6). The direct consequence will be improved quality of patient care along with scientifically aligned guidance for recipients of health care (7). To improve the moral decision making abilities and skills of nurses, they should first be aware of moral issues and pay them due attention, which can help increase their moral sensitivity. Sensitivity and moral responsibility are two factors that can help nurses tell right from wrong, and are constantly and strongly challenged in the nursing profession.

Moral sensitivity can be defined as awareness of and attention to the existence of moral values in situations wrought with conflict, and an individual’s self-awareness concerning their role and function under the circumstances (8, 9). On the other hand, moral sensitivity also refers to the ability to recognize moral issues and to choose the best response (10). This means that moral sensitivity is not just related to an individual's sense of what is right, but also to their personal experience and the capacity to recognize the importance of the topic at hand (11, 12). A reduction in moral sensitivity and diminished attention to moral issues may lead nurses to underrate these issues in their busy and stressful professional lives. As moral sensitivity plays an important role in ethical decision making, the causes and effective factors need to be carefully investigated. 

Some studies have identified nurses’ moral self-concept as being a very important factor influencing moral sensitivity, but there is not enough evidence to support this (13). Moral self-concept refers to an individual’s feelings, values and thoughts concerning moral issues (13). Higher levels of moral integrity may be attained when an individual's moral interests and perceptions of moral dilemmas are explored. Growth occurs as individuals try to find suitable moral solutions and make ethical decisions, not only because they recognize the importance of justice and altruism, but also because they perceive this to be the correct course of action (14). Kim et al. believed that self-concept plays an important role in the development of moral sensitivity and subsequently in moral decision making (13). Changes in moral self-concept cannot be obtained easily, as they require more powerful education systems, qualified educators, and an ethical health system (13).

Moral self-concept and moral sensitivity are influenced by the culture, customs and professional conditions in the society (15-17). There is a lack of studies investigating the relationship between moral self-concept and moral sensitivity, and we believe that these factors play an important role in moral decision making. Therefore, the present study was conducted to investigate moral self-concept and moral sensitivity in nurses employed in teaching hospitals affiliated with Zahedan University of Medical Sciences. Zahedan is the capital of Sistan and Baloochestan province and is a city in southeast Iran. 

## Method

This was a descriptive, cross-sectional study, and the samples consisted of nurses employed in teaching hospitals located in Zahedan City. 

Sample size was calculated based on the results of a pilot study conducted by the researcher on 89 nurses in order to determine the statistical power of the study. Subjects were chosen by simple random sampling using a chart of random digits.

Inclusion criteria consisted of having a minimum bachelor’s degree in nursing from an accredited domestic or international university, and having at least two years’ work experience in clinical settings. Research objectives were explained to the participants and they were assured that participation was voluntary and the information would remain confidential. The study commenced after the necessary permits were obtained from the Zahedan University of Medical Sciences, Imam Ali and social security hospitals.

The questionnaire consisted of three sections: the first section contained 7 questions on the subjects’ demographic information, including gender, age, marital status, work experience, type of ward, and position. The second section was Chang’s Moral Self-Concept Questionnaire (13), which consisted of 5 parts and 18 questions. Chang’s questionnaire was translated from English into standard Persian and then translated back into English, so that the two English versions could be compared with each other. The validity of this questionnaire was confirmed by 10 members of the nursing faculty of Kerman University of Medical Sciences. In order to determine its reliability, it was distributed among 20 nurses, and the internal consistency was calculated to be 0.76 through Cronbach’s alpha. The questionnaire used a Likert scale with responses ranging from 1 (never) to 5 (always). The total scores of the participants varied between 18 and 90.

The third section comprised the Moral Sensitivity Questionnaire designed in 2005 by Lutzen et al. in Sweden (8). It contained 28 questions and was a Likert type questionnaire with responses ranging from 1 (totally disagree) to 7 (totally agree). The participants’ total scores varied from a minimum of 28 to a maximum of 196 (18). In addition, this questionnaire contained six subscales including understanding the utilization of moral concepts in inter-personal relationships, improvement of patients’ autonomy, benevolence, experience of problems, moral struggles, and professional knowledge.

The validity and reliability of this questionnaire were determined in the same manner as the self-concept questionnaire, and the internal consistency of this questionnaire was calculated to be 0.85 through Cronbach’s alpha. The questionnaires were distributed on several consecutive days during three different shifts in Imam Ali and social security hospitals of Zahedan City. The data were analyzed by SPSS software version 17, and descriptive and analytical tests such as one-way ANOVA, Pearson's correlation and t-test were used.

Results

A total of 200 subjects participated in this study, and 12 variables were observed. The participating nurses were aged 32.19 years on average (SD = 5.77), and the mean of their work experience was 7.70 years (SD = 5.65). Demographic features are shown in table 1.

**Table 1:** Demographic data ofstudy participants (n = 188).

		Frequency (percent)
Gender	Male	21 (11.2)
	Female	167 (88.8)
Marital status	Single	44 (23.4)
	Married	144 (76.6)
Position	Nurse	131 (69.9)
	Managers	57 (30.4)
Type of Ward	Intensive care	59 (31.4)
	Surgery	24 (12.8)
	Internal	62 (33)
	Emergency	19 (10.1)
	Psychiatric	11 (5.9)
	Office	13 (6.9)

**Figure 1:**
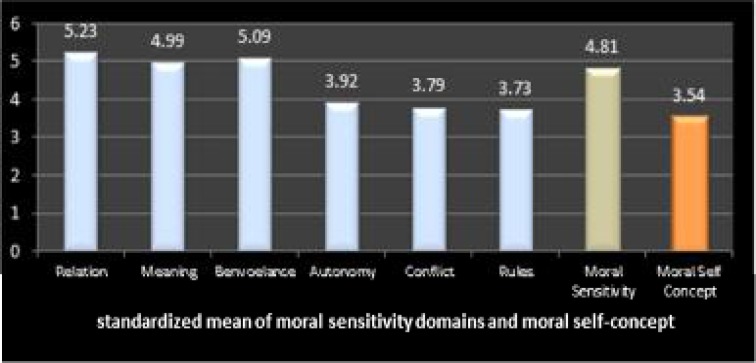
Standardized means of moral sensitivity domains and moral self-concept

As can be seen in figure 1, higher means in the various domains of moral sensitivity are related to an increased understanding of interpersonal relationships, while professional knowledge (rules) has the lowest mean.

Our findings showed that there was a positive and meaningful relationship between moral self-concept and moral sensitivity. Moreover, a significant relationship was found across all domains of sensitivity except professional knowledge. This relationship is presented in Table 2.

**Table 2:** Relationship between moral self-concept and moral sensitivity and its domains in nursing

**Components**	**R-correlation**	***P*** ** value**
Relationship	0.156	0.032*
Meaning	0.188	0.010**
Benevolence	0.234	0.001**
Autonomy	0.177	0.015*
Conflict	0.195	0.007**
Rules	0.072	0.327
Moral sensitivity	0.268	0.0001**

** *Correlation is significant at the 0.01 level*

* *Correlation is significant at the 0.05 level*

**Figure 2:**
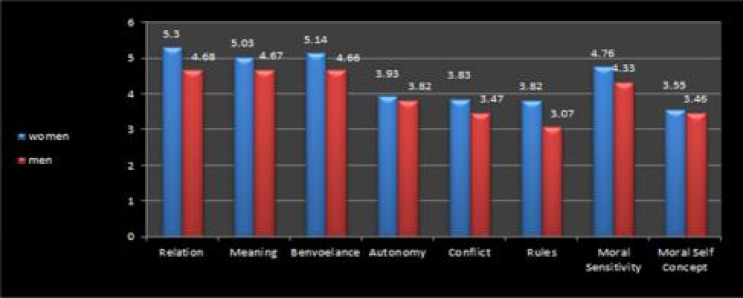
The relationship between gender and moral sensitivity and its components, and moral self-concept

In the statistical analysis carried out through a Pearson’s test, the results did not show a significant relationship between age and moral sensitivity or moral self-concept. Nevertheless, a negative and significant relationship was found across the components of moral sensitivity in the factor ‘promoting patients’ independence’, and there was a positive and significant relationship between professional knowledge (rules) and increasing age (figure 2).

The moral sensitivity of women and men was calculated by a T-test and a significant difference was found between the two groups in this respect, as women showed a higher average level of moral sensitivity. No significant difference was observed in applying moral concepts (meaning), benevolence, understanding of interpersonal relationships, and professional knowledge between men and women. Furthermore, in all of the above-mentioned domains women obtained higher average scores, except for moral self-concept, where women and men did not demonstrate a significant difference. The results are presented in table 2.

The study showed that a significant relationship existed between moral sensitivity and type of ward, but none between type of ward and self-concept. Results are shown in detail in table 3.

A Bonferroni post-hoc test demonstrated that from the viewpoint of moral sensitivity and its domains, (except for professional knowledge) the psychiatric ward produced a significant difference from the other groups. As regards moral sensitivity and the remaining domains, the score was found to be high. On the other hand, no significant difference was found between moral self-concept and professional knowledge in any of the groups.

**Table 3:** Relationship between type of ward and moral self-concept and moral sensitivity

**Components**	**Intensive Care**	**Surgery**	**Internal Medicine**	**Emergency**	**Psychiatric**	**Office**	***P*** ** value**
Relationship	5.16 (0.76)	5.14 (0.83)	5.18 (0.81)	5.20 (0.80)	6.45 (0.43)	4.98 (0.38)	0.001**
Meaning	4.97 (0.58)	4.93 (0.69)	5.10 (0.71)	4.67 (0.87)	5.53 (0.34)	5.10 (0.38)	0.033*
Benevolence	4.93 (0.73)	5.16 (0.68)	5.10 (0.61)	4.88 (0.52)	6.03 (0.44)	5.15 (0.80)	0.001**
Autonomy	3.80 (0.7)	4.02 (0.68)	3.94 (0.81)	3.92 (0.51)	4.65 (0.39)	3.53 (0.53)	0.004**
Conflict	3.54 (1.46)	3.59 (1.00)	3.80 (1.21)	4.40 (0.86)	5.18 (0.17)	3.12 (0.95)	0.001**
Rules	3.64 (0.97)	3.77 (0.79)	3.76 (0.94)	3.87 (0.87)	3.57 (0.74)	3.87 (1.17)	0.967
Moral sensitivity	4.60 (0.41)	4.75 (0.39)	4.73 (0.40)	4.63 (0.43)	5.39 (0.40)	4.64 (0.28)	0.001**
Moral self-concept	3.52 (0.34)	3.51 (0.41)	3.54 (0.34)	3.50 (0.22)	3.68 (0.14)	3.53 (0.24)	0.694

** *Correlation is significant at the 0.01 level*

* *Correlation is significant at the 0.05 level*

The relationship between work experience and other variables was investigated and no relationship was observed between work experience and moral sensitivity, or moral self-concept. Nevertheless, in the domains of moral sensitivity, such as patients’ independence and professional knowledge, there was a significant difference. 

## Discussion

The results of this study showed a positive and significant relationship between moral self-concept and moral sensitivity, and this was identical to results from a study conducted by Kim et al. on nursing managers. These findings demonstrate that an individual's concern about morality can lead to greater sensitivity and result in moral behavior at the time of decision making. Accordingly, every effort to improve nurses’ moral self-concept can lead to higher moral sensitivity and eventually better ethical conduct. Finally, promotion of moral self-concept is possible through personal effort or education (13). In the present study, moral self-concept had a positive and significant relationship with all domains of moral sensitivity except for professional knowledge. It seems that unilateral development and clinical and practical skills and knowledge do not guarantee the development of moral self-concept in nurses, and other strategies are needed to develop nurses’ moral sensitivity and moral self-concept. 

In this research the degree of nurses' moral self-concept was relatively high, with an average score of 3.52. The study by Chang et al. also confirmed these results (13). We were unable to find similar studies on the level of nurses’ moral self-concept, although one study investigated moral improvement in nurses in which they attained a score of 3.23 (19). Nevertheless, previous studies on nurses’ level of self-consciousness had produced lower scores. In Cho and Kang’s study (1984) the average score attained by students was 3.86, and in Son's research (1996) the nurses’ score was 3.76 (20). The reason for nurses’ high moral self-concept may be that nursing is a basically moral career as ethics are the essence of care (21, 22). The moralistic nature of the nursing profession is both due to the content of this major and the students’ motivations. In other words, it is safe to assume that nurses are for the most part driven by strong moral motives, and even if they are not aware of it at the beginning, the recognition is obtained in the process of professional socialization. Nurses’ moral self-concept develops in the course of their career and professional values will come to be of utmost importance. In addition, there is a high incidence of events that involve ethical considerations, and it is only expected that nurses will have higher levels of moral self-concept.

Among the questionnaire items related to professional moral self-concept, the highest score pertained to ‘I'm a person who maintains his/her honor’. In the study conducted on nursing managers in Korea, the item ‘I have no feelings of pride and joy’ gained the highest score (13). Maintaining dignity is an internal value that received the highest score in the present study due to individual and non-material values, while in the case of Korean nurses the external factors of self respect and honor may have been predominant. It is obvious that this perspective is influenced by cultural differences in the research environments. In our study the lowest score pertained to the item, ‘To surpass others I rely on unethical ways’ and in the Korean study (13, 16), the lowest score was related to the item, ‘I want others to think that I am reliable’ (13). In this respect, the influence of internal and external values in the two cultures should not be overlooked. In other words, inappropriate conduct is generally judged according to one’s conscience, but in certain cultures external values such as the perceptions of other people are more important. Although the technical skills of nursing are rather similar all around the world, the actual experience is not the same everywhere because cultures differ not only from country to country, but also from region to region. Perceptions of morality can also vary in different areas. It appears that the above-mentioned differences in scores are related to cultural differences and as discussed previously, culture is a very important factor in moral issues (16, 23).

In the present study no relationship was found between demographic features and moral self-concept. In previous studies work experience and social relationships were effective factors in moral self-concept (13), but they did not appear to be relevant in our study. This means that in our study population the subject of moral self-concept is totally independent of demographic features. In the Korean study, however, work experience and social communication were related to the level of education and also to moral self-concept, which indicates the effect of external values such as work experience and social relationships on nurses’ moral self-concept in the Korean culture.This may be due to the lower sample size in the present study than in theirs, or because their subjects were nursing managers while we carried out our study on nurses.

The results of this study showed that the moral sensitivity of nurses was higher than average at 4.71 out of a possible 7. In another study conducted on 143 nurses in Kerman this figure was 4.77 (24), and in a study by Comrie, it was 4.95 (18). This level is far from ideal and certainly not appropriate in a career so intervowen with morality. Therefore, suitable measures should be taken to increase nurses’ moral sensitivity and standards through the education system (21).

In the domain of moral sensitivity, the highest score was related to understanding the dynamics of interpersonal relationships, and the lowest pertained to professional knowledge. In a study by Abbaszadeh et al., the domain of professional knowledge had the highest score and the domain of problem experience and moral dilemmas the lowest. In another study conducted by Comrie (18), applying moralistic concepts had the highest, and problem experience and moral struggles the lowest scores. Moreover, there was no relationship between demographic features and the above-mentioned factors, which was similar to the results of the Abbaszadeh et al. study (24). Nevertheless, Kim et al. found that at the age of 25 to 30, nurses had a higher moral sensitivity compared to those younger than 25 or older than 30. Kim believed that around these ages nurses have a better understanding of nursing practices so they have greater sensitivity (25). Furthermore, in a study by Park, moral sensitivity was found to increase with age (26). On the other hand, a negative and significant relationship was observed between nurses’ ages and the domain of improvement in patients’ independence. This showed that older nurses emphasize less independence in patients and try to put patients’ requests in their later priorities. In the domain of professional knowledge there was a positive and meaningful relationship with age which was confirmed by the Abbaszadeh et al. study (2011). These findings show that organized on-the-job training in clinical work settings probably leads to the development of professional knowledge in nurses, and this emphasizes the importance of training and further education for nurses.

There was a significant relationship between gender and moral sensitivity in that women had higher levels of moral sensitivity than men. In the domains of understanding and managing interpersonal relationships, applying moralistic concepts, expression of benevolence, and professional knowledge, there was also a significant relationship with gender, with women obtaining higher averages in all of the aforementioned domains. It appears that gender is strongly related to moral sensitivity, or according to the Gilligan studies, women exhibit care morality rather than equality morality. These two forms of morality are accompanied by justice morality, and woman have a greater tendency for care, rather than equality and justice. The most important form of morality in the nursing profession is care morality, and this was confirmed by current study results, and the women obtained higher scores overall (27-29).

Between the type of ward and moral sensitivity there was a meaningful relationship, with the psychiatric ward obtaining a higher than average score. Other domains except for professional knowledge also showed a positive and meaningful relationship, and the department of psychological medicine obtained a higher than average score. Our findings demonstrated that the nurses working in specialized departments had less moral sensitivity. In Kim’s study lower moral sensitivity was observed in the ICU department. This may be due to the fact that the nurses in these units face moral problems more frequently than other units which reduces their moral sensitivity (13). In contrast, there was a lower degree of stress in the department of psychological medicine in the Imam Ali Hospital, and this may help explain the greater moral sensitivity that was observed.

There was no meaningful relationship between work experience and moral sensitivity in the nurses in this study using a Pearson’s correlation test. On the other hand, a negative relationship existed between work experience and improvement in patients’ independence, while there was a significant relationship between work experience and professional knowledge. These findings point to the relationship between patients’ independence and professional knowledge and age, and point to the correlation between these two domains and work experience, which may also result in increased professional knowledge.

## Conclusion

Nurses often encounter serious situations in their medical practice that entail legal and moral outcomes, and must therefore be fully equipped for the decision making process. They should be sensitive to the ethical issues in their profession as this can help them to respect their patients’ rights, and also to manage problematic situations and create a difference between their professional and personal values. According to the findings of this study, moral self-concept is an effective factor in moral sensitivity, and therefore suitable planning for the development of the former should be a priority for nurses. Furthermore, there is a need for more extensive studies in the field of moral self-concept and the factors contributing to it in order to evaluate the defects and find suitable solutions for emerging dilemmas. 

There were several limitations in this study including a lack of domestic and international resources on the topic of moral self-concept in nurses. Another limitation was the sample size, and it is recommended that this study be repeated with larger samples. Furthermore, nurses’ exhaustion and emotional preoccupations were among the undesirable factors that could have been removed through an explanation of the objectives of the study and combining participation with personal satisfaction. As previously stated in the introduction, moral self-concept and moral sensitivity fall under the influence of factors such as culture, traditions, and different professional conditions, and this highlights the necessity of repeating this study in different settings. 
